# Promoting Accessible Research for Children With Intellectual Disabilities; Lessons Learnt From Adaptations Through the Covid‐19 Pandemic

**DOI:** 10.1111/jir.70055

**Published:** 2025-10-03

**Authors:** Catherine Laverty, Caroline Richards

**Affiliations:** ^1^ School of Psychology University of Birmingham Birmingham UK

The intellectual disability (ID) research landscape has seen a considerable transformation over the past decade, with a new emphasis on inclusive and open research practices being prioritised (de Haas et al. 2022). Inclusivity in research is an umbrella construct that at its heart requires research to be truly collaborative, accessible and driven by the interests of the population for whom it is about (Garratt et al. 2022; Walmsley and Johnson 2003), with evidence of inclusivity in practice increasing throughout the field of ID research. Concomitant with this transformation in ID research have been paradigmatic shifts to the wider research landscape over the past 4 years, with many research studies requiring wholesale redesign to be compatible with the remote, restricted environment conferred by the Covid‐19 pandemic. Common adaptations include research studies moving online (Song et al. 2020), utilising remote technology to collate data (eye tracking—[Vos et al. 2022; Fraser et al. 2025]; video conferencing software—[Humphries et al. 2022]; mobile apps—[Wijesooriya et al. 2020]) and adopting a citizen science approach to minimise researcher contact with participants (Dow et al. 2022). We discuss how these research design adaptations, necessary for research to be compatible with a remote context, may have inadvertently had widescale benefit for children with ID for whom traditional research practices were often inaccessible. First, we will highlight adaptations necessary for ID research to be compatible with the remote context of the pandemic, before discussing how these approaches may support wider research adaptations seen across the research landscape to promote more replicable and open science. Finally, we will provide practical suggestions for ID researchers, discussing how these two approaches are compatible to promote better research practices. ID researchers designing future studies should endeavour to incorporate some of the lessons learned from research designs created within the context of the Covid‐19 pandemic to promote a more accessible and inclusive environment for people with ID overall.

Adapting research protocols to be inclusive and accessible for children with ID is not a new idea. Historically, the traditional approach has been to subtly adapt research paradigms to be more suitable for the specific needs of children with ID. Examples of such adaptations include the addition of easy‐read information sheets into existing protocols (Chinn and Homeyard 2017), transformation of ‘traditional’ psychological tasks such as the ‘tower of London’ (Masson et al. 2010) and creating alternate survey instruments specifically for people with ID (Nicolaidis et al. 2020). These subtle shifts in research design reflect a direct challenge that cuts across ID research; that of ensuring adaptations maintain task validity while making research accessible to the individual needs of children with ID. This consideration often means that though some research studies do become accessible to some children with ID, often results are not entirely generalisable to *all* children with ID. Subtle design changes are not always sufficiently accessible for people with the most severe intellectual impairments or more complex individual needs. For children who have an ID *and* a co‐occurring condition (such as autism, ADHD, epilepsy or rare genetic syndromes), this difficulty is exacerbated, and these children are consequently often excluded from research studies (Russell et al. 2019). These subtle shifts to task administration do not reduce the emotional, physical and sensory barriers implicit in travelling to participate in face‐to‐face research (often deemed the ‘gold‐standard’ of participant research). As such, while the evidence base for informed and robust research grows, so does the potential bias if this research only represents the proportion of children with ID who are able to feasibly participate.

In direct contrast to the subtle changes to research designs to be accessible for people with ID are the adaptations that were necessary for research to be compatible within the Covid‐19 pandemic. The pandemic impacted every aspect of life around the globe, from health care provision, education, the economy and in turn the day‐to‐day conducting of research (World Health Organisation 2020). Within this period, researchers were faced with a decision to either pause ‘traditional’ research designs that were incompatible with the necessary social distancing and pandemic constraints, or completely redesign and adapt plans to be suited to the new landscape. Many researchers chose to make adaptations, with common practice becoming protocols that were delivered completely online (Torrentira 2020), designs that prioritised video conferencing software rather than in‐person assessments and minimising tangible research equipment such as moving towards the use of mobile apps rather than participant paperwork (Tiersma et al. 2022). Entirely new ways of conducting direct clinical assessments were devised (e.g., the creation of the ‘BOSA’ as an alternate to the gold standard ‘ADOS’ [Dow et al. 2022]). These approaches were designed primarily to ensure research could continue within the new and restrictive environments conferred by the pandemic. Yet, it should be acknowledged that an additional, inadvertent implication of these changes was to open research participation to children for whom the traditional research environment was inaccessible. Therefore, the move to remotely delivered research may have reflected a narrowing of research methodologies but opened up research for those whose preference and only method of access would be to participate within their own, familiar contexts.

More broadly across the research landscape, the move to preregistration and open research designs (accelerated by the Covid‐19 pandemic) means that many adaptations made by researchers can be seen by all. Registration of research is both heavily encouraged and becoming more widely adopted (Simmons et al. 2021), allowing researchers to document precise plans and hypotheses *before* embarking on data collection. This process has two immediate benefits to the scientific field generally and for ID research specifically. Firstly, researchers clearly documenting original hypotheses *and* analytical approaches prior to data collection and subsequent scientific articles reduce the risk of poor research practices and increase the trustworthiness of findings. Poor research practices, such as hypothesising after results are known (‘HARKing’), searching for significant results (p‐hacking) or poorly powered analyses, can subtly infiltrate research designs, compromising the validity and reliability of findings. This is particularly concerning in clinical and rarer populations such as children with ID, where results have considerable clinical implications. Secondly, the use of preregistration allows for the wider scientific field to see and learn from current adaptations described by other researchers, conferring incremental advantages to progress and facilitating more collaborative approaches to addressing complex scientific questions. For example, Table 1 provides a nonexhaustive list of some examples of preregistrations of remote research designs for studies working with children with ID, available on the Open Science Framework [OSF] website. The number of registrations that outline research *specifically* for children with ID is increasing yearly, demonstrating an increasing propensity in ID research to adopt open and replicable research practices.

**TABLE 1:** Preregistrations of remote research designs for studies working with children with ID available on the Open Science Framework.

Title	References	Summary preregistered adaptations	Population
Evaluation of emotion recognition through human biological motion among children with typical development and children with intellectual disability.	Riviere, E., Courbois, Y., and Gentaz, E. (2023, July 3). Evaluation of emotion recognition through human biological motion among children with typical development and children with intellectual disability. https://doi.org/10.17605/OSF.IO/DJZQ8	Experimental study will be presented via a computer program.	50 children aged 4–12 years with an intellectual disability, 150 children aged 4–12 years without an intellectual disability.
A clinical checklist of causes of poor behavioural outcomes in children with moderate‐profound intellectual disability and complex needs.	Trower, H., and Crawford, H. (2022, May 23). A clinical checklist of causes of poor behavioural outcomes in children with moderate‐profound intellectual disability and complex needs. https://doi.org/10.17605/OSF.IO/TVUPW	The quantitative measures described above will be uploaded to Qualtrics so that participants can complete all measures online from home.	Parents/carers of children with moderate‐profound intellectual disability and complex needs as well as community paediatricians
Autistic children's understanding of nonverbal gestures directed to a first and third person	Elsherif, M. M., Richards, C., van Zoest, W., and Surtees, A. (2022, August 24). Autistic children's understanding of nonverbal gestures directed to a first and third person. https://doi.org/10.17605/OSF.IO/7VWCF	Exploring joint attention in a nonverbal environment. All testing will be conducted remotely, with children and parents in their homes.	30 autistic children without ID, 30 autistic children with mild–moderate ID, 30 children with mild–moderate ID and 30 neurotypical children
Using digital social contact during the COVID‐19 pandemic: Experiences of people with intellectual disabilities and their social network	Bakkum, L., Piekema, L., Adam, E., Brug, A. t., Douma, L., Embregts, p., … Tharner, A. (2022, March 11). Using digital social contact during the COVID‐19 pandemic: Experiences of people with intellectual disabilities and their social network. https://doi.org/10.17605/OSF.IO/PKBMQ	Online questionnaires and semistructured interviews	*N* = 31 individuals with (intellectual) disability living in a 24‐h long‐term care facility, age ≥ 16 years
Sleep‐Impulsivity‐Behaviour Project	Agar, G., Brown, C. N., Bagshaw, A., Devine, R. T., Symons, F., Richards, C., and Skubera, M. (2021, January 4). Sleep‐Impulsivity‐Behaviour—Study 2. https://doi.org/10.17605/OSF.IO/V9CHJ	Remote sleep assessments with autistic children who have an ID via a wrist‐worn device.	Children aged 5–19 years with ID and a formal diagnosis of autism

Finally, the use of protocols that incorporate ‘citizen science’ methods has been one key innovation that has enabled research to be particularly accessible for children with ID. Citizen science approaches can be operationalised as the involvement of nonspecialised members of the public (e.g., a child with ID's main caregiver) to work in collaboration with scientists to administer research protocols (Bonney et al. 2009). This approach was often necessary throughout the pandemic, given remote contexts were sometimes the only way to collect data compliant with social distancing restrictions. However, citizen science methods are now becoming more widely adopted, particularly as a potential solution to widespread issues of generalisability and replicability that have posed a crisis to psychological research (Yarkoni 2022). Across research with typically developing children, the citizen science approach is now well utilised, for example, parents submitting video footage of infants for researchers to study laughter (Addyman and Addyman 2013), obtaining large datasets to describe vocalisations in infancy (Semenzin et al. 2021) and exploring the value of short breaks of activity within the classroom (Booth et al. 2020). Often, these approaches bring scientific protocols *into* everyday environments. This approach, therefore, seems entirely advantageous and compatible with research aiming to be more inclusive for people with ID, yet citizen science approaches have been rarely adopted for children with ID outside of the necessary remote research context of the pandemic. Of course, the involvement of nonspecialised personnel within the scientific design confers challenges, with a lack of potential rigour and control being two of the most obvious issues (Nov et al. 2014). Yet, when considering the broader issues mentioned thus far (a lack of accessibility of traditional research designs, wider issues of generalisability and reliability of research), the inclusion of citizen science methods across ID research is one exciting area for researchers to consider to improve the accessibility and reliability of research.

Across psychological research as a whole, it is widely accepted that replicable and open research does advance science, improve theory development and enhance the robustness of research (Nosek et al. 2022). Open practices are being more commonly adopted within developmental research, with teams now documenting and publishing their practices for others to learn from (Turoman et al. 2024). Within neurodevelopmental research, journals such as ‘Autism’ have also recently expanded their article submission categories and now accept registered reports as part of a commitment to supporting open and reproducible research practices (Manning 2024). Importantly, the COVID‐19 pandemic accelerated many of these shifts by forcing the research community to reconsider how knowledge is generated, shared and accessed. Preprints, open data and remote collaboration became essential, revealing both the feasibility and necessity of more open and inclusive research practices. It is our belief that a move towards open and inclusive research practices will, and should, now follow ID research. It will be important to monitor the impact of these approaches and to evaluate the quality of the data, inclusivity and generalisability that these practices confer.

To conclude, over the last 4 years, research and daily life have adapted dramatically across the globe. The change in working restrictions has meant a necessary shift for researchers to continue their work, summarised in this article and in Figure 1. Within the field of ID research, many of these changes, as outlined within this report, have inadvertently created opportunities for more children with ID to access research. Though none of the adaptations highlighted within this article provide a complete ‘fix’ for wider issues both across the scientific field and the ID research landscape, these changes undoubtedly move ID research towards more accessible and inclusive practices, which are positive first steps. Specific adaptations made throughout the past 4 years represent real, practical changes that were necessary to ensure the viability of research, rather than subtle, incremental shifts, as seen previously. As we move towards a new ‘normal’ in terms of conducting research without the constraints seen over the past 4 years, ID research should capitalise on the changes adopted during the pandemic to (1) document adaptations in a shared, open way (e.g., through platforms such as OSF), (2) evaluate the utility and precision of such adaptations in relation to collating scientific evidence and (3) ensure lessons from adaptations are carried forward into future research designs. Table 2 presents some practical examples of ways researchers working in child ID research can incorporate open and accessible research practices into aspects of the research process. Moving towards a more open and accessible research landscape is not an all‐or‐nothing approach; small changes to the way in which we design, conduct and disseminate research will result in advances across the field. Ultimately, addressing accessibility issues *and* research issues of reliability and generalisability can go hand in hand, and the lessons learned over the last 4 years provide an important steer for next steps. Insights gained over the past 4 years have laid important groundwork and point the way forward for more inclusive and impactful ID research.

**FIGURE 1:**
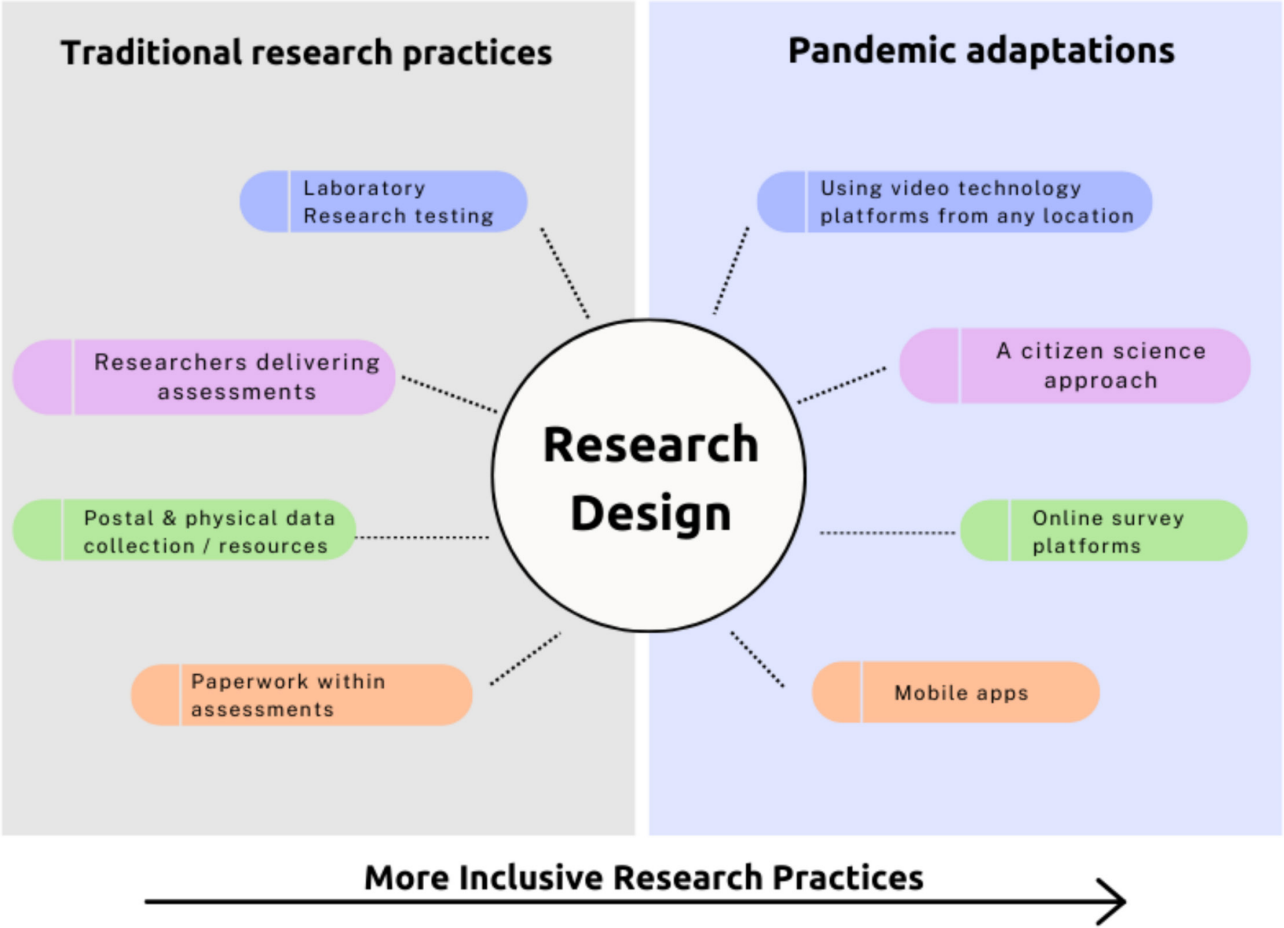
Visual representation of traditional research practices, adaptations made in the context of the Covid‐19 pandemic and the represented shift to more inclusive practices.

**TABLE 2:** Incorporating open and inclusive research practices into the research process.

Phase of research	Adaptation	Summary	More information
Design	Participant and stakeholder involvement	There is a growing understanding of the importance of including patients or members of the public in all aspects of the research process, rather than designing research ‘for’ particular populations without consultation of the appropriateness or relevance. In best practice research, patient and participatory involvement (PPI) groups of relevant personnel or stakeholders are convened at the beginning of a research study and involve themselves in decisions such as the research design, methodological processes, advertisement and dissemination of results. To reflect the continued time commitment and expertise of such groups, it is suggested that reimbursement is given for participation in such sessions. For research involving children with ID, PPI groups may include children with ID (with necessary accessibility adaptations), parents or caregivers of participants, healthcare or educational professionals or relevant support groups or charities that are relevant to the target population for whom the research is focused on. Where research involves adults with ID, adaptations should be made to ensure they are able to participate in PPI groups, should they wish to do so.	The Health Research Authority (HRA) provides detailed information on best practice principles for public involvement in research https://www.hra.nhs.uk/about‐us/news‐updates/new‐best‐practice‐guidance‐public‐involvement/ See Beighton et al. (2019 ) and Tromans et al. (2023 ) for detailed description of adaptations that can be made for people with an intellectual disability to access PPI groups.
	Preregistration of research plan	Preregistration is the process of specifying your research plan prior to the collection of data via an open repository or platform. The registration is timestamped and outlines study methods, hypotheses and analysis plans in an open way for all to see. Most importantly, the approach is flexible, meaning researchers can update the registration throughout the study to highlight the status of a project, documenting any elements of the process that have changed as they go. Processes such as preregistration prevent practices that can be harmful for ‘good science’.	The Open Science Framework (OSF—https://osf.io/) is a commonly used free platform used to preregister research studies. See Simmons et al. (2021) for a detailed description of ‘bad’ science practices preregistration prevents.
	Registered reports	Registered reports are an alternative publication method to traditional articles, where research proposals are peer‐reviewed before data collection commences. Research articles are then principally accepted for publication based upon the research question, with publication not linked to study findings.	The Centre for Open Science (COS—https://www.cos.io/initiatives/registered‐reports) has an up to date list of participating journals that accept registered reports as well as information on the process. See Chambers and Tzavella (2022) for detailed discussion of registered reports.
Data collection	Methods of data collection to increase accessibility	For children with ID, accessibility of methods is key to the success of inclusive research. Where possible, researchers should offer a flexible approach to data collection for participants, taking into consideration individual needs and challenges (see Figure 1 for a nonexhaustive summary of adaptations). PPI groups could provide a research team with invaluable input on how best to structure a study to promote inclusion and accessibility. Any insights or adaptations should be included within the dissemination of a project to benefit other research studies.	See Hewitt et al. (2023 ) for a recent review of inclusive health and care research for people with ID.
Dissemination	Accessible ways of disseminating research findings	Aside from the standard dissemination pathways (such as academic journals or conferences), researchers should endeavour to disseminate findings in an open and accessible way. Examples include infographics, lay research summaries or media such as videos or podcasts. Consulting PPI groups (mentioned previously) may highlight other meaningful dissemination pathways that would support the sharing of information with those who may find it helpful.	The charity cerebra have a range of research based parent and professional guides freely available (https://cerebra.org.uk/get‐advice‐support/parent‐guides/). See St. John et al. (2022 ) for recommendations for inclusive research for people with ID, in particular free resources in supplementary materials that could support accessible dissemination. See Parent‐Johnson and Duncan (2024) for a discussion on inclusive dissemination for research with individuals who have ID.
	Publishing journal articles open access	Where possible and funding allows, researchers should endeavour to publish research findings Open Access (OA), meaning anyone who has access to the internet could access research. Academic institutions and governing bodies now often have funds available to support open access publishing in recognition of the importance of the approach.	ResearchGate is a free platform whereby researchers may share versions of articles when they own the copyright. The site can also be used to contact authors directly, as they may be able to share research directly https://www.researchgate.net/.
	Open data	There is a growing consensus that where possible, data should be made publicly available once a research study has ended. The process has multiple benefits, ensuring transparency and accountability across the field, expanding the range of scientific questions that can be explored through pooling datasets and improving the efficiency and cost‐effectiveness of research studies. In addition, for children with ID for whom participating in data collection may be challenging, open data reduces participant burden across research projects.	The UK Data Service is a free platform for hosting data, as well as research protocols and papers linked to a research study https://ukdataservice.ac.uk/help/other‐data‐providers/open‐data‐resources/ . You can find data published by central government, local authorities and public bodies here—https://www.data.gov.uk/.
	Open measures and adaptations	Where possible, researchers should endeavour to develop free‐to‐access measures or adaptations of measures to be suitable for children with ID. Where this is not possible, a detailed summary of measures or adaptations (inclusive of reflections on the success of such adaptations) is helpful to promote inclusivity and collaboration.	The UK Data Service, OSF, research gate or paper appendices are useful places to host details of measures or adaptations.

## Conflicts of Interest

The authors declare no conflicts of interest.

## Data Availability

Data sharing not applicable to this article as no datasets were generated or analysed during the current study.

## References

[jir70055-bib-0001] Addyman, C. , and I. Addyman . 2013. “Comedy Studies: The Science of Baby Laughter.” Comedy Studies 4, no. 2: 143–153. 10.1386/cost.4.2.143_1.

[jir70055-bib-0002] Beighton, C. , C. Victor , I. M. Carey , et al. 2019. “‘I'm Sure We Made It a Better Study …’: Experiences of Adults With Intellectual Disabilities and Parent Carers of Patient and Public Involvement in a Health Research Study.” Journal of Intellectual Disabilities 23, no. 1: 78–96. 28812949 10.1177/1744629517723485 PMC6383106

[jir70055-bib-0003] Bonney R. , H. Ballard , R. Jordan . 2009. “Public Participation in Scientific Research: Defining the Field and Assessing Its Potential for Informal Science Education.” A CAISE Inquiry Group Report. https://files.eric.ed.gov/fulltext/ED519688.pdf.

[jir70055-bib-0004] Booth, J. N. , R. A. Chesham , N. E. Brooks , T. Gorely , and C. N. Moran . 2020. “A Citizen Science Study of Short Physical Activity Breaks at School: Improvements in Cognition and Wellbeing With Self‐Paced Activity.” BMC Medicine 18, no. 1: 62. 10.1186/s12916-020-01539-4. 32178667 PMC7077117

[jir70055-bib-0005] Chambers, C. D. , and L. Tzavella . 2022. “The Past, Present and Future of Registered Reports.” Nature Human Behaviour 6, no. 1: 29–42. 10.1038/s41562-021-01193-7 34782730

[jir70055-bib-0006] Chinn, D. , and C. Homeyard . 2017. “Easy Read and Accessible Information for People With Intellectual Disabilities: Is It Worth It? A Meta‐Narrative Literature Review.” Health Expectations 20, no. 6: 1189–1200. 10.1111/hex.12520. 27862757 PMC5689240

[jir70055-bib-0007] Dow, D. , A. Holbrook , C. Toolan , et al. 2022. “The Brief Observation of Symptoms of Autism (BOSA): Development of a New Adapted Assessment Measure for Remote Telehealth Administration Through COVID‐19 and Beyond.” Journal of Autism and Developmental Disorders 52, no. 12: 5383–5394. 10.1007/S10803-021-05395-W/TABLES/7. 34914016 PMC8674519

[jir70055-bib-0008] Fraser, A. , M. Jaquiery , S. U. Gattas , et al. 2025. “Automated Gaze Orientation Estimation Behavioural Research: A Feasible Alternative to Lab‐Based Eye‐Tracking for More Accessible Remote Studies.”

[jir70055-bib-0009] Garratt, D. , K. Johnson , A. Millear , S. Picken , J. Slattery , and J. Walmsley . 2022. “Celebrating Thirty Years of Inclusive Research.” Social Sciences 11, no. 9: 385. 10.3390/SOCSCI11090385.

[jir70055-bib-0010] de Haas, C. , J. Grace , J. Hope , and M. Nind . 2022. “Doing Research Inclusively: Understanding What It Means to Do Research With and Alongside People With Profound Intellectual Disabilities.” Social Sciences 11, no. 4: 159. 10.3390/SOCSCI11040159.

[jir70055-bib-0011] Hewitt, O. , P. E. Langdon , K. Tapp , and M. Larkin . 2023. “A Systematic Review and Narrative Synthesis of Inclusive Health and Social Care Research With People With Intellectual Disabilities: How Are Co‐Researchers Involved and What Are Their Experiences?” Journal of Applied Research in Intellectual Disabilities 36, no. 4: 681–701. 37002721 10.1111/jar.13100

[jir70055-bib-0012] Humphries, N. , J. P. Byrne , J. Creese , and L. McKee . 2022. “‘Today Was Probably One of the Most Challenging Workdays I've Ever Had’: Doing Remote Qualitative Research With Hospital Doctors During the COVID‐19 Pandemic.” Qualitative Health Research 32, no. 10: 1557–1573. 10.1177/10497323221106294. 35672272 PMC9184831

[jir70055-bib-0013] Manning, C. 2024. “The Introduction of Registered Reports in the Autism Journal: The What, Why and How.” Autism 28, no. 12: 2949–2952. 39377131 10.1177/13623613241285676

[jir70055-bib-0014] Masson, J. D. , D. Dagnan , and J. Evans . 2010. “Adaptation and Validation of the Tower of London Test of Planning and Problem Solving in People With Intellectual Disabilities.” Journal of Intellectual Disability Research 54, no. 5: 457–467. 10.1111/J.1365-2788.2010.01280.X. 20537051

[jir70055-bib-0015] Nicolaidis, C. , D. M. Raymaker , K. E. McDonald , et al. 2020. “Advances in Measurement Methods Creating Accessible Survey Instruments for Use With Autistic Adults and People With Intellectual Disability: Lessons Learned and Recommendations.” Autism in Adulthood 2, no. 1: 61–76. 10.1089/aut.2019.0074. 32355908 PMC7188318

[jir70055-bib-0016] Nosek, B. A. , T. E. Hardwicke , H. Moshontz , et al. 2022. “Replicability, Robustness, and Reproducibility in Psychological Science.” Annual Review of Psychology 73, no. 1: 719–748. 10.1146/annurev-psych-020821-114157 34665669

[jir70055-bib-0017] Nov, O. , O. Arazy , and D. Anderson . 2014. “Scientists@Home: What Drives the Quantity and Quality of Online Citizen Science Participation?” PLoS ONE 9, no. 4: e90375. 10.1371/JOURNAL.PONE.0090375. 24690612 PMC3972171

[jir70055-bib-0018] Parent‐Johnson, W. S. , and A. W. Duncan . 2024. “Inclusive Dissemination: Inclusive Research Dissemination With Individuals With Intellectual and Developmental Disabilities.” Inc 12, no. 1: 75–82.

[jir70055-bib-0019] Russell, G. , W. Mandy , D. Elliott , R. White , T. Pittwood , and T. Ford . 2019. “Selection Bias on Intellectual Ability in Autism Research: A Cross‐Sectional Review and Meta‐Analysis.” Molecular Autism 10: 1–10. 30867896 10.1186/s13229-019-0260-x PMC6397505

[jir70055-bib-0020] Semenzin, C. , L. Hamrick , A. Seidl , B. L. Kelleher , and A. Cristia . 2021. “Describing Vocalizations in Young Children: A Big Data Approach Through Citizen Science Annotation.” Journal of Speech, Language, and Hearing Research 64: 2401–2416. 10.1044/2021_JSLHR-20-00661. PMC8632511 34098723

[jir70055-bib-0021] Simmons, J. P. , L. D. Nelson , and U. Simonsohn . 2021. “Pre‐Registration: Why and How.” Journal of Consumer Psychology 31, no. 1: 151–162.

[jir70055-bib-0022] Song, S. Y. , C. Wang , D. L. Espelage , P. Fenning , and S. R. Jimerson . 2020. “COVID‐19 and School Psychology: Adaptations and New Directions for the Field.” School Psychology Review 49, no. 4: 431–437. 10.1080/2372966X.2020.1852852.

[jir70055-bib-0023] St. John, B. M. , E. Hickey , E. Kastern , et al. 2022. “Opening the Door to University Health Research: Recommendations for Increasing Accessibility for Individuals With Intellectual Disability.” International Journal for Equity in Health 21, no. 1: 130. 36088334 10.1186/s12939-022-01730-4 PMC9464400

[jir70055-bib-0024] Tiersma, K. , M. Reichman , P. J. Popok , et al. 2022. “The Strategies for Quantitative and Qualitative Remote Data Collection: Lessons From the COVID‐19 Pandemic.” JMIR Formative Research 2022, no. 6: E30055. 10.2196/30055. PMC9034421 35394441

[jir70055-bib-0025] Torrentira, M. C. 2020. “Online Data Collection as Adaptation in Conducting Quantitative and Qualitative Research During the Covid‐19 Pandemic.” European Journal of Education Studies 7, no. 11. 10.46827/EJES.V7I11.3336.

[jir70055-bib-0026] Tromans, S. , R. Marten , P. Jaggi , et al. 2023. “Developing a Patient and Public Involvement Training Course for People With Intellectual Disabilities: The Leicestershire Experience.” Journal of Psychosocial Rehabilitation and Mental Health 10, no. 4: 411–425.

[jir70055-bib-0032] Turoman, N. , C. Hautekiet , S. Jeanneret , B. Valentini , and N. Langerock . 2024. “Open and Reproducible Practices in Developmental Psychology Research: The Workflow of the WomCogDev Lab as an Example.” Infant and Child Development 33, no. 1: e2333.

[jir70055-bib-0027] Vos, M. , S. Minor , and G. Ramchand . 2022. “Comparing Infrared and Webcam Eye Tracking in the Visual World Paradigm.” Glossa Psycholinguistics 1, no. 1: 1–37. 10.5070/G6011131.

[jir70055-bib-0028] Walmsley, J. , and K. Johnson . 2003. Inclusive Research With People With Learning Disabilities: Past, Present and Futures—University of Bristol. Jessica Kingsley Publishers. https://research‐information.bris.ac.uk/en/publications/inclusive‐research‐with‐people‐with‐learning‐disabilities‐past‐pr.

[jir70055-bib-0029] Wijesooriya, N. R. , V. Mishra , P. L. P. Brand , and B. K. Rubin . 2020. “COVID‐19 and Telehealth, Education, and Research Adaptations.” Paediatric Respiratory Reviews 35: 38–42. 10.1016/J.PRRV.2020.06.009. 32653468 PMC7301824

[jir70055-bib-0030] World Health Organisation 2020. “WHO Coronavirus (COVID‐19) Dashboard|WHO Coronavirus (COVID‐19) Dashboard With Vaccination Data.” https://covid19.who.int/.

[jir70055-bib-0031] Yarkoni, T. 2022. “The Generalizability Crisis.” Behavioral and Brain Sciences 45: e1. 10.1017/S0140525X20001685. PMC10681374 33342451

